# Genetic immunization with Hantavirus vaccine combining expression of G2 glycoprotein and fused interleukin-2

**DOI:** 10.1186/1479-0556-6-15

**Published:** 2008-10-22

**Authors:** Huang Hao, Li Xiu, Zhang Zehua, Jia Min, Hu Hongbo, Wu Zhihong, Zhu Zhenhua, Wan Xiaohong, Huang Hanju

**Affiliations:** 1 Department of Pathogentic Biology, Tongji Medical College, Huazhong University of Science and Technology, Wuhan city 430030, PR China; 2 Department of Health, Tongji Medical College, Huazhong University of Science and Technology, Wuhan city 430030, PR China; 3 Center of Experimental Medicine, Wuhan first hospital, Wuhan city 430022, PR China

## Abstract

In this research, we developed a novel chimeric HTNV-IL-2-G2 DNA vaccine plasmid by genetically linking IL-2 gene to the G2 segment DNA and tested whether it could be a candidate vaccine. Chimeric gene was first expressed in eukaryotic expression system pcDNA3.1 (+). The HTNV-IL-2-G2 expressed a 72 kDa fusion protein in COS-7 cells. Meanwhile, the fusion protein kept the activity of its parental proteins. Furthermore, BALB/c mice were vaccinated by the chimeric gene. ELISA, cell microculture neutralization test in vitro were used to detect the humoral immune response in immunized BALB/c mice. Lymphocyte proliferation assay was used to detect the cellular immune response.- The results showed that the chimeric gene could simultaneously evoke specific antibody against G2 glycoprotein and IL-2. And the immunized mice of every group elicited neutralizing antibodies with different titers. Lymphocyte proliferation assay results showed that the stimulation indexes of splenocytes of chimeric gene to G2 and IL-2 were significantly higher than that of other groups. Our results suggest that IL-2-based HTNV G2 DNA can induce both humoral and cellular immune response specific for HTNV G2 and can be a candidate DNA vaccine for HTNV infection.

## Introduction

The Hantaan virus (HTNV) is a member of the genus Hantavirus of the family Bunyaviridae and a causative agent of hemorrhagic fever with renal syndrome (HFRS) [1,2]. More than 100,000 cases of HFRS are reported annually, with a mortality rate between 2% and 10% [3]. However, no effective vaccine has been developed to prevent this disease.

HTNV is a spherical, enveloped virus with a genome consisting of three segments of single-stranded, negative-sense RNA. The three segments are designated as large (L), medium (M), and small (S) segments that encode RNA-dependent RNA polymerase, respectively [4]]. It is indicated that the glycoprotein (GP), which was encoded by M segment, could elicit organism to produce neutralizing antibody and could protect infected animal and human body from Hantavirus lethal infection [5]. Moreover, the neutralization sites of GP mainly exist in G2 [6].

In the past few years, immunization with naked DNA has become an alternative approach for vaccination against infectious diseases. The expression vectors used for DNA vaccines usually contain the gene(s) for an antigenic portion of a virus or bacteria, under the transcriptional control of a mammalian promoter. Direct injection of the DNA into skeletal muscles results in the synthesis of proteins that subsequently stimulate the host immune system leading to both humoral and cellular immune responses specific to the expressed protein [7,8]. Recently, several published reports describe the application of DNA vaccines to examine the protective potential of several HTNV proteins [9-11]. We have previously reported that the G2 glycoprotein gene could be expressed in cells transiently and retain specific antigenicity to the Chinese Hantavirus strain H8205 (from the Epidemic Disease Research Institute, Academy of Military Medical Sciences, China), indicating that the HTNV-G2 recombinant plasmid could be used to develope DNA vaccine against Hantavirus [12].

Use of cytokines as adjutants can enhance various immune responses when administered during the development of an immune response to a particular antigen. IL-2 is one of the extensively studied cytokine adjuvants [13-15]. When administered in multiple injections, IL-2 increased the development of antigen-specific immune response and protective immunity against challenge with the infectious agents [16]. The adjuvant efficacy was further enhanced by physically linking IL-2 to an antigen so that the cytokine effect is retained in the local environment where the immune response is initiated. Previous studies have shown that co-expression of IL-2 has also been shown to enhance the immune response to the HSV1 glycoprotein D antigen in DNA vaccines [17]. These investigations have made IL-2 an attractive adjuvant for vaccine development.

In this study, we developed a HTNV-G2 and IL-2 fusion transgene that directly elicited specific anti-HTNV humoral and cellular immune response. These results suggest that HTNV-IL-2-G2 DNA may be used as a candidate vaccine.

## Methods

### Mice, viruses, and cells

C57BL/6 mice aged 6–8 weeks- (The Laboratory Animal Center, Tongji Medical College, Huazhong University of Science and Technology, Wuhan, China) were housed in microisolated, pathogen-free facility. All experiments were carried out in accordance with the National Institute of Health Guide for the Care and Use of Laboratory Animals (NIH Publications No. 80-23, revised 1978). All efforts were made to minimize animal suffering, reduce the number of animals used, and utilize alternatives to in vivo techniques, when available. HTNV strains H8205 (Epidemic disease Research Institute, Academy of military medical sciences, China) were propagated in Vero E6 cells (VeroE6, GDC015, China Center for Type Culture Collection, China). Transient expression experiments were performed with COS-7 cells (COS-7, GDC054, China Center for Type Culture Collection, China). All cell types were maintained in Dulbecco's modified Eagle's medium (DMEM) (Invitrogen™ Life Technologies)supplemented with 10% fetal calf serum (FCS).

### Construction of vaccine plasmids

To construct the eukaryotic expression plasmid DNA vector for IL-2 DNA, the full-length IL-2 DNA was amplified from plasmid PUC19-IL-2 (Department of molecular biology, Tongji Medical College, Huazhong University of Science and Technology, Wuhan, China) encoding human IL-2 gene by PCR using IL-2 primers (forward, 5'- GGCATCGC**AAGCTT**ATGGCACCTACTTCAA-3'reverse, 5'- GCTCTCC**GGTACC**CTGCAGTGTTGAGATGA -3'), which also introduced an Hind III and KpnIrestriction sites, respectively (underlined) to the amplicons. The PCR-amplified IL-2 DNA fragments were digested with restriction endonucleases Hind III and KpnI and annealed by ligation with T4 DNA ligase (TaKaRa, Japan) to Hind III and KpnI-digested pcDNA3.1 (+) expression vector (Invitrogen™ Life Technologies) DNA, downstream of the CMV promoter, hereafter referred to as pcDNA3.1/IL-2.

To construct the DNA vector containing the fused form of HTNV IL-2-G2, HTNV G2 DNA was amplified from plasmid pcDNA3.1/HTNV-G2 (Department of Pathogenic Biology, Tongji Medical College, Huazhong University of Science and Technology, Wuhan, China) encoding G2 gene by PCR using primers(forward, 5'- GG**GGTACC**TACGGGCTGCAAGTGCTTCTGAAAC -3'reverse, 5'- CCG**CTCGAG**TAGGACTATGCCTTCTTGTGC -3') designed to introduce KpnI and XhIrestriction sites at 5'and 3'ends of the amplicons, respectively(underlined), and subcloned into pcDNA3.1/IL-2 at the KpnI restriction site's 3'-flank to the IL-2 gene, resulting in pcDNA3.1/HTNV-IL-2-G2. Following ligation, the reconstruct plasmids were introduced into transformation-competent E. coli DH5α (TaKaRa, Japan) and cultured overnight. Plasmid DNA was purified by using Qiagen Maxiprep DNA purification kits(Qiagen, San Diego, CA) according to the manufacturer's directions. The presence of the inserted DNA fragment was confirmed by restriction enzyme digestion and gel electrophoresis. All the constructs were further verified by DNA-sequencing (Applied Biosystems, USA). The DNA was finally resuspended in phosphate-buffered saline (PBS) at a concentration of 1 mg/ml.

### Cell culture and transfection

COS-7 cells were grown in DMEM medium supplemented with 10% calf serum. Lipofectamine™ 2000 reagent (Invitrogen™ Life Technologies) was used in transfection. The transfection was performed according to the protocol of Invitrogen. COS-7 cells were transfected with pcDNA3.1/HTNV IL-2-G2 and the control group pcDNA3.1(+). After 48–72 h, the expressed proteins were detected.

### Immunofluorescence

For immunofluorescence, the transfected COS-7 cells were fixed in PBS containing 4% paraformaldehyde for 15 min at RT, followed by extensive washing with PBS. After blocking in PBS containing 3% bovine serum albumin (BSA) for 1 h, the cells were incubated with anti-G2 Mab (Epidemic disease Research Institute, Academy of military medical sciences, China) or polyclonal mouse anti-IL2 antibody (Santa Cruz, CA, USA, at 1:50 dilution) for 1 h at RT, bridged by avidin-conjugated anti-mouse IgG (Santa Cruz, CA, USA, at 1:300 dilution) for 1 h at RT, subsequently detected with FITC-labeled goat anti-mouse IgG (Santa Cruz, CA, USA, at 1:32 dilution) and rodamin-labeled biotin (Santa Cruz, CA, USA, at 1:100 dilution) for 1 h at RT. After thoroughly washing, the coverslips were mounted and observed with a confocal fluorescence microscope

### Western blot analysis

To detect protein expression by western blot, the transfected cells were lysed with lysis buffer. The cell lysates were run on sodium dodecyl sulfate-polyacrylamide gel electrophoresis (SDS-PAGE), and then transferred electrophoretically to a nitrocellulose membrane. The membranes were incubated with monoclonal antibody (MAb) against IL-2 (Santa Cruz, CA, USA, at 1:50 dilution) or against HTNV G2 (at 1:400 dilution), detected by horseradish peroxidase (HRP)-conjugated goat anti-mouse IgG (at 1:600 dilution, Santa Cruz, CA, USA), and developed using a chemiluminescent substrate (Pierce, Rockford, IL, USA). The emitted light was captured on X-ray film.

### Recombinant protein production

The cells cultured at 37°C in a shaking incubator overnight. When the optical density at 600 nm (OD600) reached 0.4–0.6, a final concentration of 0.3 mM of IPTG was added to the culture to induce the protein expression, and the cells were grown for another 5 h. The cultures were harvested by centrifugation in 50 ml volumes at 5000 × g for 5 min at 4°C, disrupted by sonication in a lysis buffer containing nonionic detergent and lysozyme. IL-2-G2 proteins were purified using nickel nitriloacetic acid (Ni-NTA)-agarose column (Qiagen, Valencia, CA, USA) as recommended by the manufacturer. The identity of the purified protein was verified by western blot with anti-IL-2 MAb or anti-G2 MAb.

### DNA vaccination of C57BL/6 mice

C57BL/6 mice were immunized with DNA constructs, using a modification of a previously described method [18]. Briefly, mice, three to four per group, five groups in total, were immunized with 100 μg of pcDNA3.1/HTNV-IL-2, pcDNA3.1/HTNV-G2, pcDNA3.1/HTNV-IL-2-G2, pcDNA3.1 (+) plasmids (in 50 μl of PBS) and PBS respectively, per mouse in both anterior tibial muscles that had been pretreated with 0.25% bupivacaine 2 days before vaccination. The second immunizations were given at intervals of approximately 2 weeks using the same amount of DNA, followed by boost once with 200 pM per mouse of their homologous recombinant proteins 2 weeks after the second DNA immunization.

### Detection of serum HTNV G2 and IL-2 specific antibodies

Sera were collected from a retroorbital plexus puncture 10 days after last protein boost or an animal's tail vein puncture of immunized mice at weeks 0, 2, 4, 6, 8, 10, 12, 14 and 16 (where 0 represents the first day of immunization). The G2 and IL-2-specific antibodies in the sera were determined by an indirect ELISA method. The purified G2 of HTNV and IL-2 protein(Epidemic disease Research Institute, Academy of military medical sciences, China) were used as coating antigen. Then the sera were serially diluted and reacted with G2 and IL-2 antigen. HRP-SPA conjugate was used as secondary antibody. The titers were defined as the reciprocal of the positive highest serum dilution.

### Cell microculture neutralization test

This test was performed on Vero E6 cell monolayers in 96-well tissue culture plates with HTNV strains H8205. The E6 clone of Vero cells was grown in DMEM medium supplemented with 10% calf serum. The sera titer were twofold serially diluted from 1:15 in DMEM medium containing 2% fetal calf serum and filtrated through 0.22 μm filter. Then it were incubated with viruses at 37°C for 90 min. Virus-sera mixtures were applied to cell monolayers and incubated at 37°C for 8–10 days in 5% CO2 incubator. Thereafter, the cells were lysed with three consecutive freeze-thaw cycles. HTNV antigen in the lysate was detected by sandwich ELISA. The anti-G2 Mab were used as coating antibody and HRP-SPA conjugate were used as secondary antibody.

### Lymphocyte proliferation assay

Eight weeks after the final booster dose, mice were sacrificed. Spleens were removed from immunized mice and purified by lymphocytes separation medium. Then the mice splenocytes suspension were applied in 96-well tissue culture plates and co-incubated for 68 h with HTNV G2 and IL-2 antigen, which was responsible for induction of cellular response to HTNV, and blank control at 37°C in 5% CO_2 _incubator. After applied MTT [(4,5-dimethylthiazole-2-yl)-2,5-diphenyl tetrazolium-bromide,5 mg/ml] 20 μL/well for 4 h, 150 μL/well DMSO was used to dissolve the blue formazun precipitate. The extinction coefficient was measured at 490 nm. The proliferation index was calculated as follows: proliferation index = *A490 nm *value stimulated with antigen/*A490 nm *value stimulated with blank control.

### Statistical analysis

Differences between assays are analyzed using one-tailed or two-tailed pair t-test as appropriate (GraphPad Prism). Probability values of ≦ 0.05 are considered to represent significant differences.

## Results

### Construction and expression of vaccine plasmids in vitro

**Figure 1:**
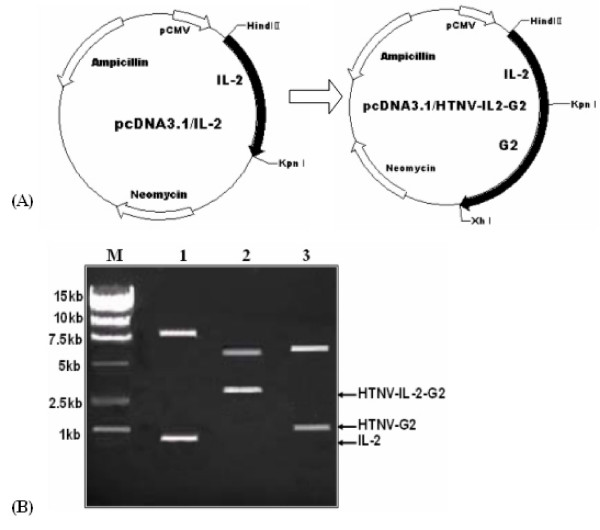
**Construction and expression of vaccine plasmids**. (A) A schematic representation of the recombinant vaccine plasmids. The plasmid pcDNA3.1/IL-2 was constructed by inserting the HindIII and an KpnIdigested the PCR-amplified IL-2 DNA fragment into HindIII and an KpnI sites of pcDNA3.1 (+) between the cytomegalovirus promoter sequence and the bovine growth hormone polyadenylation sequence. The plasmid pcDNA3.1/HTNV-IL-2-G2 was constructed by inserting the KpnI and XhIdigested HTNV G2 DNA fragment into the KpnI and EcoRI sites of pcDNA3.1/IL-2 plasmid upstream and in frame with the IL-2 gene. (B) Identification of recombinant plasmids by using restriction enzyme digestion and agarose gel electrophoresis. Lane M, DNA molecular size marker; lanes1–3, pcDNA3.1/HTNV-IL-2-G2 digested with HindIII and KpnI, HindIII and XhI, KpnI and XhI, respectively.

The eukaryotic expression plasmids, pcDNA3.1/HTNV-IL-2-G2 were constructed as described in Fig. 1A. By restriction analysis, a 0.8, 1.9 or 2.7 kb fragment was identified, respectively, in HTNV IL-2-G2 recombinant plasmid (Fig. 1B), which corresponded, respectively, to IL-2, HTNV G2 or fused HTNV IL-2-G2 DNA. The nucleotide sequences introduced into vectors were further confirmed by automatic sequencing machine (Applied Biosystems, USA), and matched human IL-2 (accession number NM_000586) sequences deposited in the GenBank, perfectly (data not shown).

### Expression of vaccine plasmids in vitro

**Figure 2:**
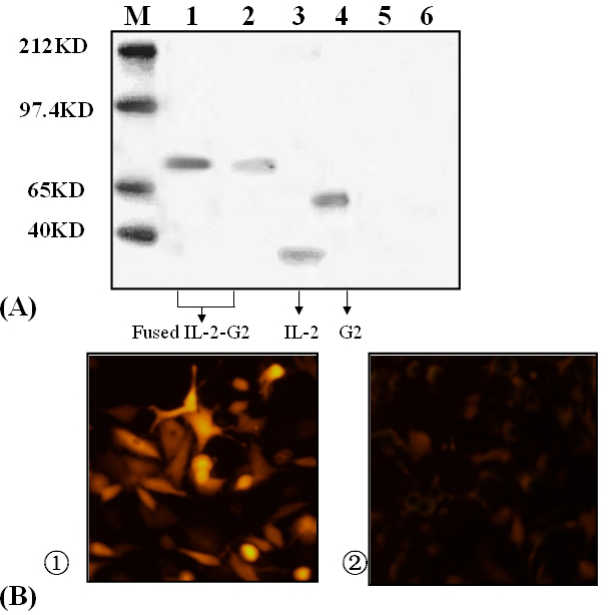
**Genetic immunization of vaccine plasmids**. (A)Western blot analysis of protein expression of vaccine DNA in transfected COS-7 cells. Lane 1 and 2, lysate of transfected COS-7 cells with pcDNA3.1/HTNV-IL-2-G2 were incubated with mouse anti-IL-2 and anti-G2 Mab, Lane 3 and 4, lysate of IL-2 and G2 protein with mouse anti-IL-2 antibody and mouse anti-G2 Mab, respectively. Lane 5 and 6, lysate of transfected COS-7 cells with pcDNA3.1(+)were incubated with mouse anti-IL-2 antibody and mouse anti-G2 Mab, respectively. M, molecular weight marker proteins: the mobility of size standards (in kDa) is shown to the left. (B) Indirect immunofluorescence of COS-7 cells transfected by recombinant vector pcDNA3.1/HTNV-IL-2-G2 (B-1) and pcDNA3.1(+) (B-2), incubated with mouse anti-G2 Mab, bridged by avidin-conjugated anti-goat IgG, and detected by rodamin conjugated biotin and FITC conjugated anti-mouse IgG, followed by laser confocal scanning.

We analysed the expressions of expected proteins by western blot. As shown in Fig. 2A, the immunoblots showed a large protein band of about 72 kDa present in COS-7 cells transfected with pcDNA3.1/HTNV-IL2-G2 that reacted with both anti-IL2 and anti-G2 antibodies. This corresponds to the expressed 72 kDa G2-IL2 fusion protein.

Immunofluorent staining revealed cytoplasmic expression of IL-2-G2 in COS-7 cells transfected with pcDNA3.1/HTNV-IL-2-G2 (Fig. 2B). These results indicated that the vaccine plasmidDNAs could express efficiently in eukaryotic cells.

### Genetic immunization results of vaccine plasmids

To investigate whether the recombinant DNA vectors could elicit HTNV G2-specific humoral response, ELISA and cell microculture neutralization test in vitro were used to detect the humoral immune response in immunized BALB/c mice. Meanwhile, the stimulation index of splenocytes to G2 was measured by MTT assay.

**Figure 3:**
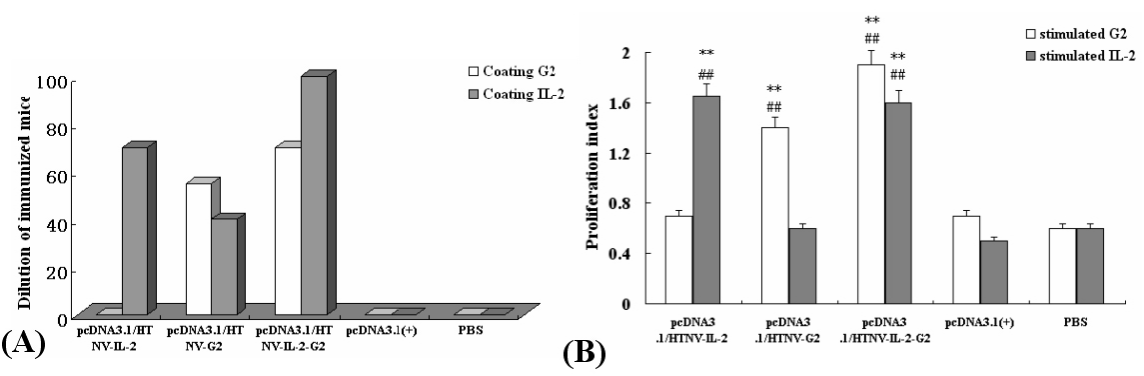
**Antibody response in the sera of C57BL/6 mice immunized with different recombinant DNA vaccines**. C57BL/6 mice were primed twice with pcDNA3.1/HTNV-IL-2, pcDNA3.1/HTNV-G2, pcDNA3.1/HTNV-IL-2-G2 and pcDNA3.1 (+) and PBS plasmids respectively. The second immunizations were given at intervals of approximately 2 weeks using the same amount of DNA. A) ELISA results of average titers of immunized mice sera. B) Analysis of the stimulation index of splenocytes of immunized mice to HTNV G2 and IL-2. All data were obtained from three independent experiments. Error bars represent means ± SEM. Significantly different from the corresponding control and vector. (**P < 0.01, vs pcDNA3.1(+). ##P < 0.01, vs PBS group).

ELISA results showed that the average titer of the specific antibody against HTNV G2 in the mice immunized with G2-IL2 was 1:70. Whereas, the specific antibody of the average titer of pcDNA3.1/HTNV-IL-2-G2 group against IL-2 was 1:100. The specific antibody of the average titer of pcDNA3.1/HTNV-IL-2 group against HTNV G2 and IL-2 were negative and 1:70. The results of pcDNA3.1/HTNV-G2 group against HTNV G2 and IL-2 were 1:55 and 1:40. The results of pcDNA3.1 and PBS groups against HTNV G2 and IL-2 were all negative (Fig. 3A).

Cell microculture neutralization test results showed that part of the immunized mice of pcDNA3.1/HTNV-IL-2-G2 group could elicit neutralizing antibodies with different titers. But the neutralizing antibody titers were low (Table 1).

**Table 1:** The detection of neutralizing antibody titers in the sera of immunized mice

Group of animals	Animals No.	Neutralizing antibody titers
pcDNA3.1/HTNV-IL-2 immunized mice	1.1	1:10

	1.2	-

	1.3	-

	1.4	-

pcDNA3.1/HTNV-G2 immunized mice	2.1	1:10

	2.2	1:20

	2.3	-

	2.4	1:10

pcDNA3.1/HTNV-IL-2-G2 immunized mice	3.1	1:40

	3.2	1:20

	3.3	1:10

	3.4	1:10

pcDNA3.1 (+)immunized mice	4.1	-

	4.2	-

	4.3	-

	4.4	-

PBS immunized mice	5.1	-

	5.2	-

	5.3	-

	5.4	-

Lymphocyte proliferation assay results showed that the stimulation indexes of splenocytes of pcDNA3.1/HTNV-IL-2-G2 group to IL-2 and G2 were all significantly higher than that of other groups (Fig. 3B).

## Discussion

It is well known that the G1 and G2 of HTNV may both play important roles in evoking neutralizing antibody for protecting against HTNV infection and in cell-mediated protective immune response. Cytokines as indicators and regulators of the immune network play important roles in the immune and inflammatory responses. Previous studies showed the potential of the IL-2 gene as a molecular adjuvant, which appear promising for protective immunity against virus infection [19,20]. Meanwhile, augmentation of DNA vaccine-elicited immune responses using plasmid IL-2 has been reported in several murine disease models [21,22].

In the present study, we developed a novel chimeric HTNV IL2-G2 DNA vaccine by genetically linking IL2 gene to the full-length G2 segment DNA. Then we investigated the expression of the recombinant vectors in mammalian cells. RT-PCR and western blot analysis results proved by mRNA and protein levels that the recombinant vector was transiently expressed in COS-7 cells-. The immunofluorescence results showed that pcDNA3.1/HTNV-IL-2-G2 DNA could express protein in mammal cells.

Previous studies demonstrated that DNA vaccine plasmids expressing HTNV glycoproteins could elicit the production of G2 protein in mice, hamsters and nonhuman primates. In vaccinated hamsters, for example, the presence of G2 antigen correlated with protection against HTNV infection [23]. On the basis of this work, BALB/c mice were vaccinated with the recombinant eukaryotic expression vector, pcDNA3.1/HTNV-G2-IL2. To avoid the potential side effects associated with systemic administration of recombinant cytokines, such as the generation of antibodies to IL2 and the neutralization of endogenous IL2, a sustained but low level of cytokines delivered to tissues of immune interactions may reduce the toxicity of these pleiotropic compounds, while improving their therapeutic and practical value in providing vaccine adjuvant effects. Direct injection into mouse skeletal muscle of expression vector encoding cytokines provides such a means. ELISA and cell microculture neutralization test in vitro were used to detect the humoral immune response in immunized BALB/c mice. The stimulation indexes of splenocytes to IL2 and G2 were measured by lymphocyte proliferation assay.- ELISA results showed that the chimeric gene could simultaneously elicit specific antibody against IL-2 and G2.

Mice neutralizing antibodies protect mice and humans from viral infection, it is very important to use a chimeric vaccine such as the G2-IL2 that elicits a strong immune response in vivo. Cell microculture test results showed that the mice in the pcDNA3.1/HTNV-G2-IL2 vaccination group produced different neutralizing antibody titers; but they were all consistently low. This result may be caused by variations in the expression level of genetic vaccines that elicit low levels of antibody production

It has been demonstrated that both humoral and cellular immune response were important in host defense against HTNV [24]. Adoptive transfer of immune T cells protected suckling mice from death following infection with HTNV [25]. Furthermore, it was also found that T cells expressing CD4-CD8^+ ^markers on their surface were especially important for elimination of infectious viruses in vivo [25]. Others reported that T-cell-mediated immunity plays an important role in resistance of mice to HTNV infection. Recently, CTL epitopes in HTNV have been identified [26]. MTT results showed that the stimulation indexes of splenocytes of chimeric gene group to G2 and IL-2 were significantly higher than that of control. The chimeric genes could also evoke cellular immune response in mice.

In conclusion, the present results demonstrate that a DNA vaccine that fuses IL2 with HTNV G2 can directly elicit a specific anti-HTNV humoral and cellular immune response in BALB/c mice. It was suggested that IL-2 and HTNV G2-based HTNV DNA strategy appears to be an attractive approach for the rational design and development of new and more efficacious DNA vaccines for HTNV-associated diseases.

## Competing interests

The authors declare that they have no competing interests.

## Authors' contributions

HH carried out the molecular genetic studies, participated in the sequence alignment and drafted the manuscript. LX, ZZ, JM and HH carried out the immunoassays. WZ participated in the sequence alignment. ZZ and WX participated in the design of the study and performed the statistical analysis. HH conceived of the study, and participated in its design and coordination. All authors read and approved the final manuscript.
